# Serum ferritin concentration in early pregnancy and risk of subsequent development of gestational diabetes: A prospective study

**Published:** 2017-03

**Authors:** Sedigheh Soheilykhah, Mahdieh Mojibian, Maryam Jannati Moghadam

**Affiliations:** 1 *Endocrine Department, Shahid Sadoughi University of Medical Sciences, Yazd, Iran.*; 2 *Department of Obstetrics and Gynecology, Shahid Sadoughi University of Medical Sciences, Yazd, Iran.*; 3 *Department of Obstetrics and Gynecology, Mojibian Hospital, Yazd, Iran.*

**Keywords:** Ferritin, Gestational Diabetes

## Abstract

**Background:**

Elevated serum ferritin concentration is associated with insulin resistance and diabetes. Recently it has also been described in gestational diabetes mellitus (GDM).

**Objective:**

A prospective study was done to determine whether there was a relationship between serum ferritin concentration in early pregnancy and the risk of GDM.

**Materials and Methods:**

A study was performed on 1,384 pregnant women with gestational age of 12-16 weeks. A blood sample was obtained for measurement of ferritin in the first trimester. Diagnosis of GDM was done by 75 gr oral glucose tolerance test between 24-28 wk.

**Results:**

Women who developed GDM had a higher concentration of serum ferritin than women who did not develop GDM (p=0.01). A ferritin concentration of 45 ng/ml was calculated to be the 75^th^ percentile for healthy pregnant women. Considering this level 32% in the GDM group and 25.2%of normal subjects exhibited high ferritin levels (p=0.01). The risk of GDM with these high levels of ferritin was 1.4-fold higher than that for subjects with lower concentrations. The Odds Ratio was 1.4 (95% CI= 1-1.87) (p=0.01). After adjusted for age Odds Ratio was 1.38 (95% CI=1.02-1.86) (p=0.03) and after adjustment for pre-pregnancy Body Mass index, the adjusted odds ratio was 1.31 (CI= 0.96-1.79) (p=0.08). After multivariable adjustment (age and body mass index), the adjusted odds ratio was 1.3 (0.95-1.8) (p=0.09).

**Conclusion:**

High serum ferritin can be regarded as a significant risk factor for the development of gestational diabetes.

## Introduction

Ferritin, the major iron storage protein, has a function in iron metabolism (1, 2). Serum ferritin concentration displays the measure of body iron stores because it is highly correlated with bone marrow iron (3, 4). High serum ferritin levels have been demonstrated in many chronic disorders and vascular inflammation (5-9). Mildly elevated body iron stores have been associated with elevations in glucose homeostasis indexes (10). A significant correlation between higher serum ferritin levels and insulin resistance syndrome has been showed (11-13). 

Some studies revealed a significant association between higher serum ferritin and risk of type 2 diabetes (14, 15). Nevertheless, there is a discrepancy in data about whether increased serum ferritin is an independent risk factor for diabetes and whether higher levels follow inflammation or increased iron pools (7, 8, 12). Some data have demonstrated that iron excess may have a role in insulin resistance at the cellular level (16, 10). Greater levels of ferritin in women with impaired glucose tolerance test and GDM have been shown in epidemiologic studies (17, 18). 

The goal of this cohort study was to determine the relationship between serum ferritin concentration and risk of GDM.

## Materials and methods

A prospective study was done on pregnant women with 12-16 wk gestational age. These subjects were recruited from two prenatal clinics (Mojibian and Shahid Sadoughi Hospitals) in Yazd, Iran between Jan 2010 and Feb 2013. Pregnant women were excluded from the study because of serious non-obstetric problems such as type 1 or 2 diabetes, malignancies, acute or chronic inflammatory or infective diseases, acute or chronic liver disease and iron deficiency anemia. General information including maternal age, the level of education, reproductive medical histories was obtained from the subjects’ medical records; Height was measured at entry to prenatal clinic, and pre-pregnancy BMI was obtained by re-calling pre-pregnancy weight in Kilogram divided by the square of height in meters. 

These data were considered as covariates in the analysis. At 12-16 wk of pregnancy fasting 5ml venous blood samples were taken for measurement of hemoglobin, ferritin, iron, and total iron-binding capacity (TIBC). Blood samples were stored at -70^o^C up to assayed. Prenatal iron supplementation was continued by all the participants based on the national policy, and all women received the same dosage (50 mg of elemental iron/day) after the fourth month of pregnancy. Assessment of gestational diabetes was done by 75 gr oral glucose tolerance test between 24-28 wk of pregnancy. The oral glucose tolerance test was performed after an overnight fast of 8-14 hr while the subjects were on an unrestricted diet with unlimited physical activity for at least three days. Plasma glucose measured at fasting and 1 and 2 hr after 75 gr glucose load. Women were diagnosed with GDM if at least 1/3 diagnostic criteria were met (fasting plasma glucose ≥92 mg/d, 1- and 2-hr plasma glucose levels of ≥180 mg/d and ≥153 mg/d, respectively) (19). Glucose was measured by the photometric method (Pars Azmun Kit, Iran). Intra-assay and inter-assay coefficient variation of glucose were 1.74% and 1.19% respectively.

Serum ferritin was measured by ELISA method using two high-affinity monoclonal antibodies in an immune metric assay system (Delaware Biotech Kit, USA). The Intra-assay and inter-assay coefficient variation of ferritin were 5.7% and 6.6% respectively. Iron was measured by the spectrophotometry method using the Bio system Iron Ferrozin Kit code 11509 (Bio system S.A., Costa Brava 30, Barcelona (Spain). The Intra-assay and inter-assay coefficient variation were 2.2% and 2.9%, respectively. Iron binding capacity was measured by the spectrophotometry method using the Bio system Kit (Bio system S.A., Costa Brava, 30.08030 Barcelona (Spain). Hb was assayed by the ELISA method (Sigma Diagnostics, St. Louis, MO), and hematocrit (HCT) was measured by micro hematocrit capillaries.

**Ethical consideration**

This study was approved by Shahid Sadoughi University of Medical Science Ethical Committees. All subjects gave written Informed consent for participation in the study.

**Statistical analysis**

Univariate statistics were considered for continuous variables, and a ^2^ test was used for categorical variables. Data of continuous variables were expressed as mean±SD, and because ferritin levels were not normally distributed, the results were shown as medians (interquartile range). For the assessment of correlation between ferritin and risk of GDM the Spearman correlation was used. Multiple linear regression analyses were used to determine the relations between elevated serum ferritin (independent variable, coded as 75 percentile versus lower percentile) and pre-pregnancy BMI and age that were the dependent variables. 

The logistic regression analyses were performed to find the effect of high serum ferritin on the risk of GDM. Odds ratios (ORs), adjusted odds ratios (AORs) and their 95% CIs from the logistic regression coefficients and corresponding covariance matrices, and the P for trend were computed. Potential confounding variables known to be associated with GDM were included in multivariable models (maternal age and pre-pregnancy BMI) were tested in separate models because they correlated with serum ferritin. All statistical analyses were performed using statistical package for the social sciences, version 17.0, spss Inc Chicago, Illinois, USA. 

## Results

In this study from 1,384 subjects, 1,358 subjects performed OGTT at 24-28 weeks of pregnancy, and 26 women (1.6%) did not follow our study (OGTT was not done or referred to another center). The characteristics of participants are shown in two groups (Table I). Women who developed GDM during pregnancy (22.1%) were older (p<0.0001) and had higher pre-pregnancy BMIs (p<0.0001) and higher serum ferritin concentrations than women who did not develop GDM (Table I).

The mean serum iron concentration was 111.5±58.3 µg/dl in the GDM and 98.6±41.7 µg/dl in the normal group; no significant difference was found. Total iron binding capacity was 344±72.5 µg/dl in the normal and 334±56 µg/dl in the GDM group, which was not statistically significant. The serum level of hemoglobin was not significantly different between the two groups. In pregnant women with gestational diabetes, the serum ferritin level was found to be higher in comparison with healthy pregnant women and the difference was statistically significant (p=0.001). The linear relationship between serum ferritin with maternal age was 0.05 (p=0.06) and with pre-pregnancy BMI was 0.1 (p=0.001). 

A ferritin concentration of 45 was calculated to be the 75^th^ percentile for healthy pregnant women. Considering this level as the cut-off point to define high ferritin, 32% in the GDM group and 25.2% of normal subjects exhibited high ferritin levels (p=0.01). The risk of having GDM with these high levels of ferritin was calculated to be 1.4-fold higher than that in subjects with lower ferritin concentrations (OR 1.4 95% CI= 1-1.87) (p=0.01) (Table II). We further examined the association of high serum ferritin at entry and pre-pregnancy BMI on the risk of GDM. In pregnant women with serum ferritin level more than 45 ng/ml and pre-pregnancy BMI ≥30, the risk of GDM was 2.37 (95% CI=1.39-4.04) (Table III). After adjustment with age adjusted odds ratio (AOR) was 1.38 (95% CI=1.02-1.86) (p=0.03) and after adjustment with pre pregnancy BMI AOR was 1.31 (CI= 0.96-1.79) (p=0.08). After multivariable adjustment (age and BMI), the AOR was 1.3 (0.95-1.8) (p=0.09) (Table IV). Our study showed after adjustment with pre-pregnancy BMI the risk of GDM in pregnant women with ferritin concentration more than 45 ng/ml was moderately modified. Also, we did not find any association between high hemoglobin level, Hb >13.5 g/dl (75^th^ percentile), and risk of GDM with OR=1.19 (95% CI 0.88-1.6) (p=0.27). The sample size of our study was sufficient because it had 90% power to detect a 40% increase in the risk of GDM in pregnant women who had ferritin concentrations more than 45 ng/ml in the first trimester.

**Table I:** Characteristics of pregnant women in the two groups

Variables	**GDM **	**NON GDM**	**p-value**
Pre pregnancy BMI (kg/m2)	26.4±5	24.7±4.4	0.001
AGE ( years)	29.4±5.4	27.1±4.5	0.001
Ferritin ( ng/ml )	41±35 MD=35(29)	35.5±30.730.7(26.2)	0.01
Fe ( µg/dl )	111.5±58.3	98.6±41.7	0.16
TIBC( ( µg/dl )	334±56	344±72.5	0.43
Hb (g/dl)	12.7±1.1	12.7±3.5	0.7

Data of continuous variables were expressed as mean ± standard deviation, and because ferritin levels were not normally distributed, the results were showed as medians (interquartile range) χ^2^ test was used for categorical variables

**Table II:** Association between elevated serum ferritin and risk of GDM

**Serum ferritin**	**GDM (N=281) [n(%)]**	**Normal (N=998) [n(%)]**	**p-value**	**OR ( 95% CI)**
<45 ng/ml	191 (%68)	747 (%74.8)	0.02	1.4(1.05-1.87)
≥45 ng/ml	90 (%32)	251 (%25.2)	0.02	

The logistic regression analyses were performed to find the effect of high serum ferritin on the risk of GDM

**Table III:** Association between elevated serum ferritin and BMI on the risk of GDM

**Serum ferritin**	**GDM (N=281)**	**Normal (N=998)**	**p-value**	**OR ( 95% CI)**
Ferritin <45and BMI <30	21.4%	78.6%	0.02	2.37(1.39-4.04)
Ferritin ≥45and BMI ≥30	39.3%	60.7%	0.02	

**Table IV:** Regression analyses of ferritin and other confounding variables to predict GDM in the pregnant women

**Dependent Variable**	**Independent variables**	**OR**	**CI**	**p-value**
GDM	Ferritin, Age	1.38	1.03-1.86	0.03
GDM	Ferritin, BMI, <30 reference, >30	1.31	0.96-1.79	0.08
GDM	Ferritin, Age, BMI, <30 reference, >30	1.3	o.95-1.8	0.09

## Discussion

Our study demonstrated that pregnant women with elevated serum ferritin concentration in early pregnancy had a higher rate of gestational diabetes. Our results were in concordance with Chen which showed that women with the highest quintile of serum ferritin had a twofold increased risk of GDM (17). Shimin and colleagues in a systematic review revealed that increased risk of GDM is significantly associated with greater levels of ferritin and also risk of GDM may be related to higher levels of hem iron (20). Sharifi found that “serum ferritin levels were highly associated with GDM independently of BMI” (21). 

Zein and co- workers in a prospective observational study on 104 pregnant women, observed that ferritin level in early pregnancy was significantly correlated to glucose level after OGTT at 1-h and 2-h (22). Lao *and colleagues *showed that among pregnant Chinese women, the mean serum ferritin concentrations at 28-30 weeks of gestation increased significantly in women with impaired glucose tolerance and patients with GDM compared with control subjects (18). Amiri showed that high ferritin levels (greater than 80 ng/ml) increased the risk of gestational diabetes to 2.4-fold [95% CI= 2.4( 0.83-6.9)] p=0.01, while in group with ferritin levels less than 20 ng/ml, the risk of GD was reduced to 82% [OR=0.8 with 95% CI (0.08-0.37) (p=0.001)] (23). Our study also demonstrated that the 75 percentile of serum ferritin was correlated with pre-pregnancy BMI. Chen also showed this relationship (17). 

Our result is similar to Amiri and colleagues which showed no significant difference was observed regarding of the serum iron and transferrin iron binding capacity between GDM and normal pregnant women; nevertheless, the serum ferritin level was higher in gestational diabetes in comparison with the normal group (23). Scholl showed that over ten years, women with high ferritin levels are more vulnerable to type II diabetes (nearly three times more ), without being associated with other risk factors including BMI, age and race (24). 

In addition, Jiang revealed that risk of type 2 diabetes is increased when the level of ferritin is elevated and this association is independent of other diabetes risk factors in healthy women (12). Chen showed a 3.5 folds increase risk of GDM in obese women that had higher level of serum ferritin (17). Our data showed that the relation between high serum ferritin levels and risk of GDM remained positive but was slightly modified after adjustment for pre-pregnancy BMI and age. After adjustment for pre-pregnancy BMI, in pregnant women with BMI ≥30 kg/m^2^, the risk of GDM in subjects with serum ferritin levels more than 45 was 1.31 (CI= 0.96-1.79) (p=0.08). 

Lao *et al* found that hemoglobin more than 13 g/dl) in pregnant women was an independent risk for GDM and that women with iron deficiency anemia had a reduced risk of GDM (16, 25). We did not find any association between high hemoglobin level and risk of GDM with OR=1.19 (95% CI 0.88-1.6) (p=0.27) Elevated serum ferritin concentration, which is associated with insulin resistance and diabetes in the general population, has also been recently described in gestational diabetes (8, 9, 25, 26). In some studies, high iron level has been shown to be a harmful factor for the body via oxidative stress and free radicals (27, 28). 

Extra iron and oxidative stress affect the pathogenesis and increase the risk of type II diabetes and other associated diseases. Recently, it has been suggested that iron has impacts on glucose metabolism even if there is no excess iron. The studies revealed that body iron stores are involved in impaired glucose tolerance and gestational diabetes since iron compounds can change insulin synthesis and secretion, increase lipid oxidation, decrease in glucose transport into the muscle and elevation in gluconeogenesis, therefore insulin resistance in tissues developed (18, 23, 29). Iron has a role in diabetes development by three mechanisms: decreased insulin production, increased resistance to insulin, and hepatic dysfunction of glucose metabolism even in the absence of excess iron (30).

Our study has some limitations. This study measured ferritin once during pregnancy and did not assess the effect of inflammatory markers on GDM.

## Conclusion

In this cohort study, we showed associations between elevations in serum ferritin levels during early gestation and the risk of GDM in pregnant women. Therefore, high ferritin can be regarded as a significant risk factor for the development of gestational diabetes.
